# An Atypical Cause of Pneumatosis Intestinalis: Norovirus Infection in an Elderly Male

**DOI:** 10.7759/cureus.81300

**Published:** 2025-03-27

**Authors:** Desiree Brionne Dillard, Cornel Popescu, Derek Zell, Nathan G Rasmussen, Cody Petrie, Lauren B Querin, Douglas Rappaport

**Affiliations:** 1 Medical School, Mayo Clinic Alix School of Medicine, Phoenix, USA; 2 Sandra Day O'Connor College of Law, Arizona State University, Phoenix, USA; 3 Emergency Medicine, Creighton University School of Medicine, Phoenix, USA; 4 Medical School, Rocky Mountain College, Billings, USA; 5 Emergency Medicine, Mayo Clinic Arizona, Phoenix, USA

**Keywords:** emergency medicine, norovirus, pneumatosis intestinalis, sepsis, viral gastroenteritis

## Abstract

Norovirus is a major cause of gastroenteritis, commonly presenting with nausea, vomiting, diarrhea, myalgia, and fever. Most cases are self-limited, but serious complications can occur, particularly in high-risk populations. Pneumatosis intestinalis is an uncommon but serious condition that may require surgical intervention and is characterized by the presence of gas in the bowel wall. Pneumatosis intestinalis is a known, albeit extremely rare, complication of norovirus infection. We report a case of norovirus gastroenteritis in a 77-year-old male complicated by septic shock, pneumatosis intestinalis, and gastrointestinal bleeding, highlighting a rare but serious manifestation of this common infection.

## Introduction

Norovirus is a leading cause of viral gastroenteritis globally [1]. Norovirus, a member of the Caliciviridae family, exhibits genomic variations impacting human health. The GII.4 genotype, in particular, has been responsible for 70%-80% of acute cases of gastroenteritis worldwide [2]. Typically, the majority of cases occur in outbreaks during the winter period [1]. Norovirus spreads primarily through the fecal-oral route, airborne droplets, and contaminated surfaces [3]. Norovirus has a short incubation period of 12-72 hours, with maximal shedding in stool occurring in the first 24-48 hours following symptom onset [1]. Norovirus affects all age demographics, with the highest incidence in pediatric populations and a significant impact on geriatric populations [1,4]. Clinical symptoms most commonly involve nausea, vomiting, and diarrhea along with nonspecific myalgias and fevers [1]. Infection is typically self-limited, although complications of severe disease, such as hypovolemic and septic shock, do occur, particularly in high-risk patient populations. Although extremely rare, pneumatosis intestinalis has been reported as a complication of norovirus [5-8].

Pneumatosis intestinalis is characterized by gas-filled areas in the intestinal submucosa and subserosa. Pneumatosis intestinalis was first described by DuVernoi in 1783 and later subclassified by Koss in 1952 [9]. It is classified into primary or idiopathic type (15%) and secondary type (85%) caused by various predisposing factors [10]. Ultimately considered a multifactorial disease with multiple predisposing factors, it can occur anywhere in the gastrointestinal tract distal to the stomach. In a retrospective review of 97 patients, the location of pneumatosis intestinalis was 46% in the colon, 27% in the small intestine, 5% in the stomach, and 7% in both the small and large intestines [11]. Management of pneumatosis intestinalis depends on the underlying etiology as well as the patient’s clinical picture. Patients exhibiting signs of shock, hemodynamic instability, or signs of peritonitis on abdominal exam will frequently require emergent surgical evaluation and intervention, including bowel resection [12]. On the other end of the spectrum, patients with clear clinical stability and benign abdominal exams can frequently be managed conservatively with observation and supportive care [13].

## Case presentation

We present a case of a 77-year-old male who presented to the emergency department with nausea, vomiting, and non-bloody diarrhea for two days. He also endorsed shortness of breath and abdominal distension. The patient’s medical history was significant for a prior aortic valve replacement on long-term anticoagulation with apixaban. He had no recent antibiotic use but had several family members at home with similar symptoms. Upon initial presentation, the patient was tachycardic with a heart rate of 114, tachypneic with a respiratory rate of 25, and had a blood pressure of 64/45. Physical examination also showed respiratory distress, abdominal distension, and abdominal tenderness with no guarding or rebound tenderness. These findings were concerning for a shock state, and the patient was started on IV fluids and symptomatic treatment with ondansetron while labs and imaging were collected.

Laboratory analysis showed an elevated lactate, elevated creatinine, and elevated blood urea nitrogen with a low bicarbonate. The leukocyte count was unremarkable (Table 1).

**Table 1:** Patient laboratory values

Laboratory Test	Patient Value	Reference Values
Lactate	11.6 mmol/L	0.5-2.2 mmol/L
Creatinine	1.87 mg/dL	0.75-1.35 mg/dL
Bicarbonate	14 mmol/L	22-29 mmol/L
Blood Urea Nitrogen (BUN)	68 mg/dL	8.0-24.0 mg/dL
Leukocytes	3.9 x 10(9)/L	3.4-9.6 x 10(9)/L
Hemoglobin	13.3 g/dL	13.2-16.6 g/dL
Platelets	267 x10(9)/L	135-317 x10(9)/L

The stool polymerase chain reaction (PCR) pathogen panel was positive for norovirus. Testing for respiratory pathogens, including COVID-19, respiratory syncytial virus (RSV), and influenza A and B, was negative.

**Figure 1:**
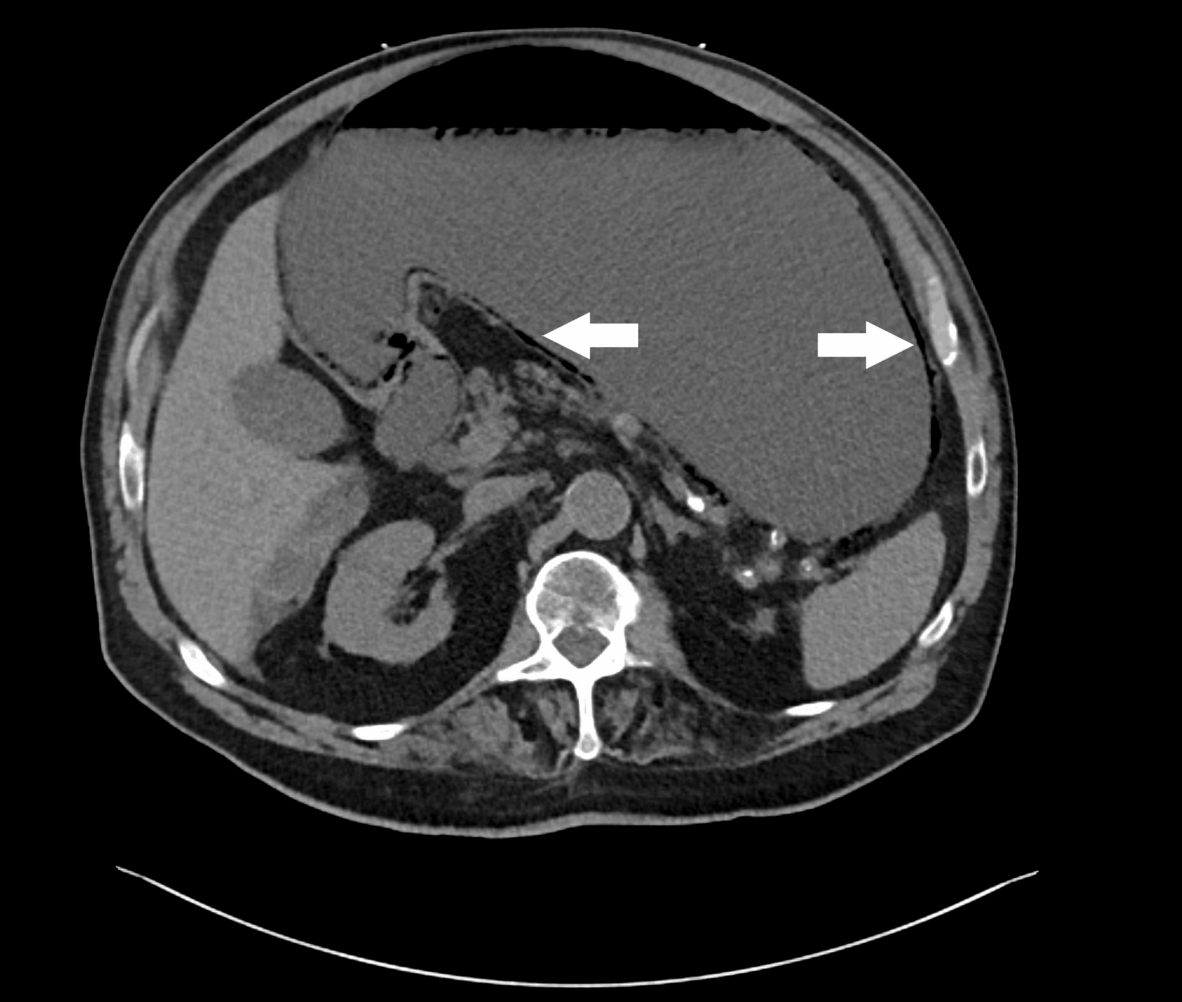
CT abdomen demonstrating gastric pneumatosis Hypodense air in the wall of the stomach is highlighted by the arrows.

**Figure 2:**
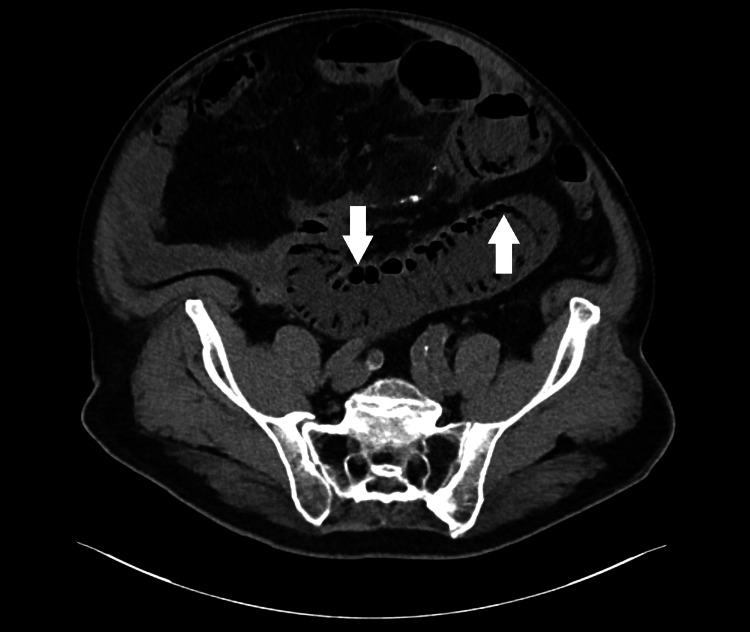
CT abdomen demonstrating pneumatosis intestinalis Hypodense air within the walls of the bowel is highlighted by the arrows.

**Figure 3:**
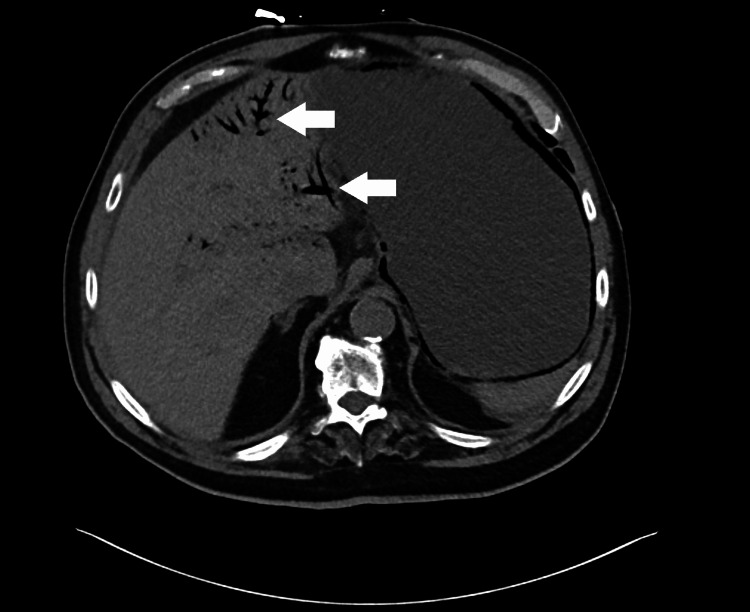
CT abdomen demonstrating portal venous gas Hypodense air present within the portal venous system is highlighted by the arrows.

Abdominal and pelvic CT revealed pneumatosis of the lower esophagus, stomach (Figure 1), and much of the small bowel (Figure 2) with associated portal venous gas (Figure 3) as well as significant gastric distension and stranding in the mesentery with associated fat necrosis, representing mesenteric panniculitis.

The patient was given ongoing intravenous fluid resuscitation, broad-spectrum antibiotics, and ultimately required vasopressor support to maintain adequate hemodynamics. A nasogastric tube was placed due to significant gastric distension, and returned 400 milliliters of dark coffee ground material. His anticoagulation was reversed with prothrombin complex concentrate over concern for gastrointestinal hemorrhage secondary to bowel ischemia. Following a discussion between the patient and our general surgery colleagues, the decision was made not to pursue emergent surgical intervention as the bowel was deemed unsalvageable if ischemic, given the extent of the pneumatosis, and the patient was admitted to the intensive care unit for management.

After his initial stabilization in the ICU with further fluids and vasopressors, the remainder of our patient’s hospital stay was complicated by a prolonged ileus that necessitated total parenteral nutrition, and he was discharged 13 days after initial presentation to the emergency department with resolution of symptoms.

## Discussion

While geographically ubiquitous, norovirus has a significant impact within the United States, causing vast illness, morbidity, and mortality with an estimated 19-21 million cases of illness annually. Norovirus accounts for 56,000-71,000 hospitalizations, 400,000 emergency department visits, 1.7-1.9 million outpatient visits, and 570-800 deaths annually [1]. Between 2014 and 2016, norovirus incidence was 5.5 cases per 1,000 person-years, highest in children (20.4/1,000) and adults ≥65 years (4.5/1,000) [1]. Outbreaks occur most commonly in crowded or heavily populated spaces, such as restaurants, health care facilities, schools and childcare facilities, municipal water sources, prisons, and military quarters [1]. It can also be associated with foodborne disease outbreaks as well [1].

Typical clinical features of symptomatic norovirus infection include nausea and non-bloody vomiting, non-bloody diarrhea, and abdominal pain [1]. The most common strain, GII.4, is associated with higher rates of hospitalization and mortality secondary to more severe disease [1]. Severe disease tends to manifest in “older adults, children <12 months, and among immunocompromised patients” [1]. The patient aligns with this population, as he is an older adult who experienced severe disease after presenting with nausea, vomiting, diarrhea, and abdominal pain. Severe norovirus-induced disease is evidenced by his arrival in a shock state, complicated by pneumatosis intestinalis.

Limited research exists on the association between norovirus gastroenteritis, pneumatosis intestinalis, and bowel necrosis/perforation. Kim et al. described four immunocompromised pediatric patients with pneumatosis intestinalis who tested positive for norovirus [6]. Another reported case involved a 30-month-old male with idiopathic nephrotic syndrome of childhood who was immunosuppressed on cyclophosphamide and steroids with a recent case of norovirus who later developed pneumatosis intestinalis [7]. We were able to find one report of norovirus-associated pneumatosis intestinalis in an adult, a 47-year-old female presenting with abdominal pain and diarrhea who tested positive for norovirus with findings of pneumatosis intestinalis on imaging [8]. This patient, like ours, was not taking immunosuppressive medication or otherwise immunocompromised. Unlike our patient, she underwent surgical treatment with resection of her cecum, ascending colon, and transverse colon, including the splenic flexure, due to colonic ischemia. All these patients survived. Our case is unique as it involves an immunocompetent adult patient who developed pneumatosis intestinalis secondary to norovirus infection and was managed medically with a resolution of symptoms.

According to the mechanical hypothesis for the pathophysiology of pneumatosis intestinalis, gas dissects into the intestinal wall from either the luminal surface (through breaches in the mucosa) or through the serosal surface (by tracking along mesenteric blood vessels) [14]. Once inside the bowel wall, gas can advance along the mesentery to distant locations [14]. In our patient, severe hypovolemia secondary to gastrointestinal losses from vomiting and diarrhea may have led to decreased perfusion to the bowel, thus leading to ischemia. Once ischemia develops in the bowel wall, bacterial overgrowth and translocation can occur, resulting in pneumatosis intestinalis and access of gut bacteria to systemic circulation, resulting in bacteremia and sepsis [15].

While there is no specific antimicrobial therapy indicated for norovirus infection, once the downstream impacts of severe hypovolemic shock progress to bowel ischemia and sepsis, early broad-spectrum antibiotic intervention and fluid resuscitation become essential [16]. In our patient with hemodynamic instability, early aggressive intravenous fluids, early antibiotic administration, vasopressor support, and appropriate imaging and surgical consultation were the keys to successful management in the acute setting.

## Conclusions

Norovirus is a very common cause of gastroenteritis worldwide. The vast majority of norovirus infections are self-limited and resolve without complication. Pneumatosis intestinalis secondary to bowel ischemia in the setting of norovirus gastroenteritis is a rare albeit described complication. The pathophysiology of bowel ischemia is thought to be secondary to bowel hypoperfusion. Bowel hypoperfusion develops as a result of a shock state from either distributive shock secondary to sepsis and/or hypovolemic shock as a result of volume losses from vomiting and diarrhea that are common in norovirus infection. Most cases described have been among immunocompromised children, so this case in an otherwise immunocompetent adult is remarkable. In patients with clinical signs of shock in the setting of gastrointestinal illness, bowel ischemia must be considered, and pneumatosis intestinalis on cross-sectional imaging may be a clue to suggest this diagnosis. Management of bowel ischemia involves aggressive fluid resuscitation, broad-spectrum antimicrobials, vasopressor support, and surgical consultation for possible bowel resection.
